# Nationwide Five-Year Official Surveillance of Microbiological Hazards in Retail Food in Poland

**DOI:** 10.3390/foods15162902

**Published:** 2026-08-19

**Authors:** Joanna Kowalska, Elżbieta Maćkiw

**Affiliations:** Department of Food Safety and Health Risk Assessment, National Institute of Public Health NIH-National Research Institute (NIPH NIH-NRI), 24 Chocimska Str., 00-791 Warsaw, Poland; jkowalska@pzh.gov.pl

**Keywords:** microbiology food safety, retail, pathogen occurrence, safety criteria, microbiological contamination, pathogens

## Abstract

This study presents the results of a completed nationwide, five-year official surveillance programme (2016–2020) conducted in Poland to assess microbiological hazards in retail food products. The programme combined official food control and national monitoring activities targeting major foodborne pathogens and hygiene indicators of public health relevance. Compliance with both EU microbiological criteria and national guidelines was assessed for *Salmonella* spp., *Listeria monocytogenes*, *Escherichia coli* (including Shiga toxin-producing *E. coli*), *Campylobacter* spp., and coagulase-positive staphylococci. The programme covered all 16 administrative regions of Poland and included 380,314 retail food samples representing major food categories. Analyses were performed in accredited laboratories using ISO standard methods. Isolates obtained from positive samples were confirmed by the National Reference Laboratory (NIPH NIH-NRI), ensuring high data reliability and consistency. The findings provide a comprehensive and nationally representative assessment of microbiological compliance in retail food over a complete five-year surveillance cycle. The dataset represents one of the largest officially generated microbiological surveillance collections of retail food in Europe, providing robust evidence for risk-based food safety management and regulatory decision-making. Food categories requiring targeted control measures were identified.

## 1. Introduction

Ensuring the microbiological quality of food in Poland is a key element of the food safety system, which includes both preventive and control activities. This process is based on European Union regulations, international standards, and Polish regulations. The microbiological quality of food in Poland is based on an integrated approach that includes regulations (e.g., Regulation [EC] No. 2073/2005) [1], food safety management systems (HACCP, GMP, GHP) and regular inspections. The contamination of food by pathogenic microorganisms or their metabolic products (toxins) poses a significant risk of foodborne illness, endangering human health [2,3,4]. In Poland, food safety is guaranteed through various measures, including the microbiological testing of retail food products. These tests verify compliance with microbiological criteria established to ensure food safety. The regulation includes food safety criteria and process hygiene criteria. According to Regulation (EC) No. 2073/2005, a “microbiological criteria” is “a criteria defining the acceptability of a product, a batch of foodstuffs or a process, based on the absence, presence or number of microorganisms and/or on the quantity of their toxins/metabolites, per unit(s) of weight, volume, surface area or batch” and “food safety criterion means a criterion defining the acceptability of a product or a batch of foodstuffs applicable to products placed on the market”. The process hygiene criteria monitor microorganism levels during production, which allows us to assess whether the technological process is correct [1]. Where the microbiological criteria laid down in Regulation (EC) No. 2073/2005 do not apply to a foodstuff, or where the product is not covered therein, the safety of the food placed on the market shall be assessed on the basis of Good Manufacturing Practice (GMP), Good Hygiene Practice (GHP), and procedures based on HACCP principles, including, where appropriate, risk assessment.

Where pathogenic microorganisms are detected, or where elevated microbiological counts may indicate a potential risk to public health in Official Control, we should assess the safety of the food and take appropriate corrective measures in accordance with applicable EU and national food law. Whenever pathogens, toxins, or relevant metabolites are detected, a risk assessment shall be conducted in accordance with applicable national and EU legislation and guidance. Where the assessment identifies a risk to public health, appropriate risk management measures shall be implemented, including withdrawal or recall of the product from the market.

Despite the established microbiological criteria and routine food safety controls, comprehensive data on the microbiological quality of food products available at retail in Poland remain limited. Information integrating the occurrence of major foodborne pathogens across different food categories and their compliance with criteria and national guidelines is scarce. Such data is essential for identifying food categories associated with microbiological non-compliance and for supporting risk-based food safety surveillance.

## 2. Objective

This study aimed to analyze official national food surveillance data from Poland to assess the microbiological compliance of retail food products with EU microbiological criteria and national guidelines. The analysis focused on *Salmonella* spp., *L. monocytogenes*, *E. coli* (including Shiga toxin-producing *E. coli*), *Campylobacter* spp., and coagulase-positive staphylococci (including *S. aureus*), as these microorganisms represent important foodborne hazards covered by the national food safety surveillance system and were consistently represented in the available surveillance dataset. Using these data, we aimed to characterize temporal trends in microbiological non-compliance and to identify the food matrices most frequently associated with non-compliant results for each pathogen, thereby providing evidence on patterns of microbiological food safety at the retail level in Poland.

## 3. Materials and Method

### 3.1. Monitoring and Official Control Studies

Studies were conducted in all sixteen provinces in Poland over a 5-year period (2016–2020) based on plans prepared by the Department of Food Safety and Health Risk Assessment of the National Institute of Public Health NIH-National Research Institute (NIPH NIH-NRI). Food samples were collected randomly. Five samples from each batch were collected for microbiological analysis. The sampling plan included all 16 administrative regions (voivodeships) of Poland, with the number of samples allocated to each region proportional to the size of its population.

A total of 380,314 food samples were collected from both small and large retail outlets as part of the planned monitoring and official control tested in total from categories: “Meat other than poultry, offal, and meat products” (25,357 samples), “Poultry meat, offal, poultry products, eggs, and egg products” (23,594), “Fish, seafood, and their products” (14,478), “Milk and milk products” (82,241), “Cereal grains and cereal-flour products” (4478), “Confectionery and pastry products” (82,963), “Nuts, including peanut’” (21), “Vegetables, including legumes” (31,485), “Fruits” (20,816), “Mineral waters and nonalcoholic beverages” (6658), “Vegetable fats” (30), “Oilseed grains” (769), “Food concentrates” (231), “Mayonnaise, mustard, sauces” (286), “Herbs, spices” (19,153), “Coffee, tea, cocoa, fruit teas and herbal” (1281), “Delicatessen and culinary products” (29,225), “Foodstuffs for special dietary purposes” (27,719), “Novel foods” (503), “Dietary supplements” (5821), “Others” (3205).

The samples were tested at the accredited laboratories of the Sanitary and Epidemiological Stations, which are located in all 16 administrative regions (voivodeships) of Poland. The analysis was performed in accordance with the following:

- Presence of *Salmonella* spp. in 25 g (culture method with biochemical and serological confirmation, PN-EN ISO 6579-1) [5,6]
- Presence of *L. monocytogenes* in 25 g (culture method with biochemical and microscopic confirmation, PN-EN ISO 11290-1) [7,8]
- Number of *L. monocytogenes* (plate method/surface plating, PN-EN ISO 11290-2) [9,10]
- Presence and identification of Shiga toxin-producing *E. coli* (STEC) (real-time method, ISO/TS 13136:2012) [11]
- Number of coagulase-positive staphylococci (including *S. aureus*) (plate method/pour plating, PN-EN ISO 6888-2) [12]
- Presence of *Campylobacter* spp. in 10 g (culture method with biochemical and microscopic confirmation, PN-EN ISO 10272-1) [13,14]
- Number of β-glucuronidase-positive *E. coli* (plate method/pour plating, PN-ISO 16649-2) [15]

Strains isolated from monitoring and official control samples were sent to our laboratory at NIPH NIH-NRI for confirmation and further studies. Samples were disqualified according to Commission Regulation (EC) No. 2073/2005 of 15 November 2005 on microbiological criteria for foodstuffs (the latest consolidated version). We adopted criteria from the national guideline for risk analysis [1].

### 3.2. Statistical Analysis

Statistical analysis was performed using R software (Vienna, Austria; version 4.5.1). The prevalence of disqualified samples was calculated as the number of disqualified samples divided by the total number of tested samples. Overall prevalence, as well as prevalence by year and product group, was expressed with 95% confidence intervals (95% CI) calculated using the Wilson method. Differences in the proportion of disqualified samples between years and among product groups were assessed using Pearson’s chi-square test. When expected cell counts were low, *p*-values were estimated using Monte Carlo’s simulation (100,000 replicates). Temporal trends were evaluated using binomial logistic regression based on aggregated data, with year included as a continuous predictor and the number of disqualified and compliant samples used as the response variable. The effect of year was expressed as odds ratios (ORs) with 95% confidence intervals (95% CI). Differences between product groups were further evaluated using bias-reduced logistic regression. Only product groups with at least 100 tested samples were included in the regression analysis. All statistical tests were two-sided, and differences were considered statistically significant at *p* < 0.05.

## 4. Results

**Figure 1:**
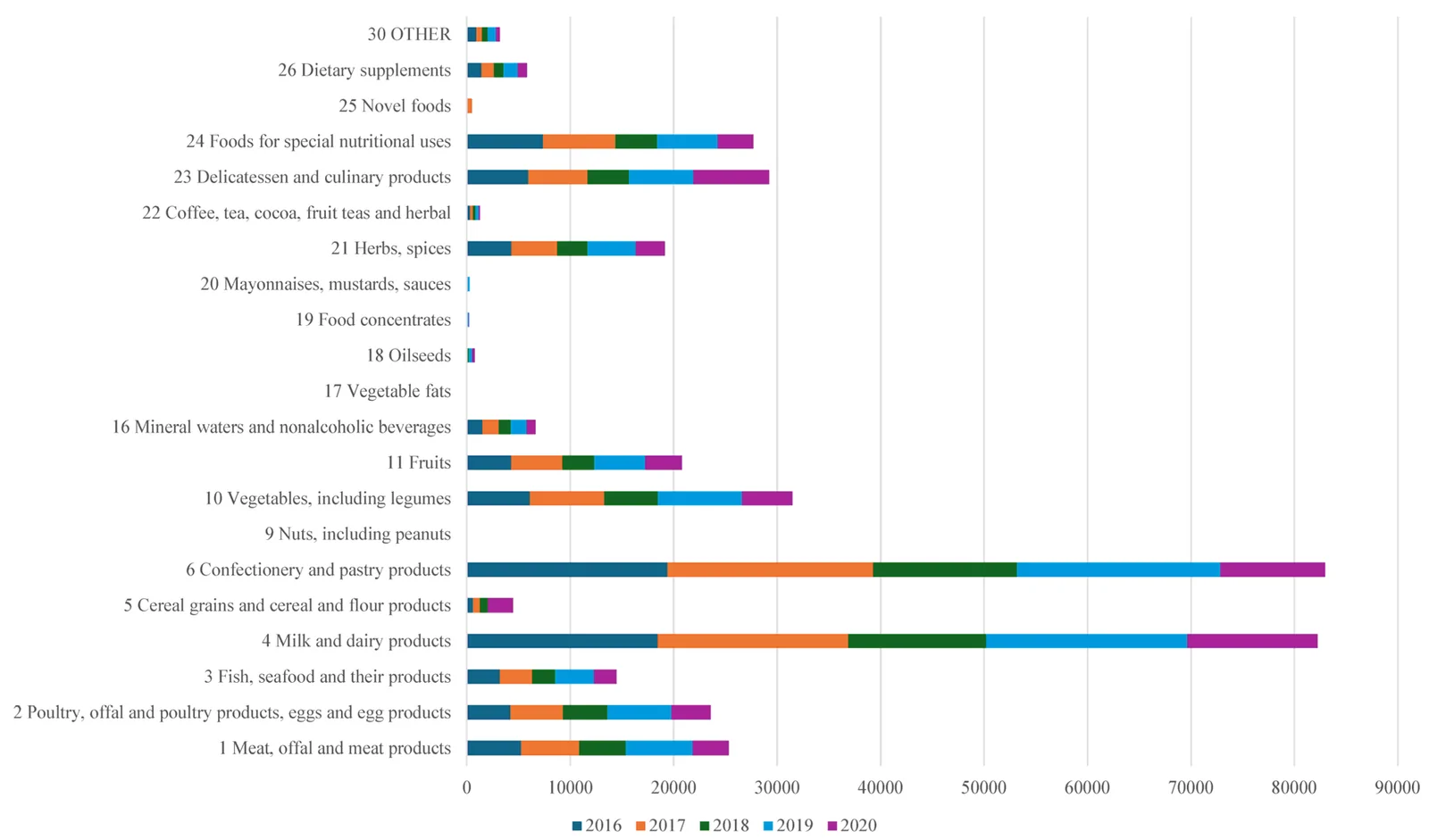
Tested samples by product groups (2016–2020).

**Figure 2:**
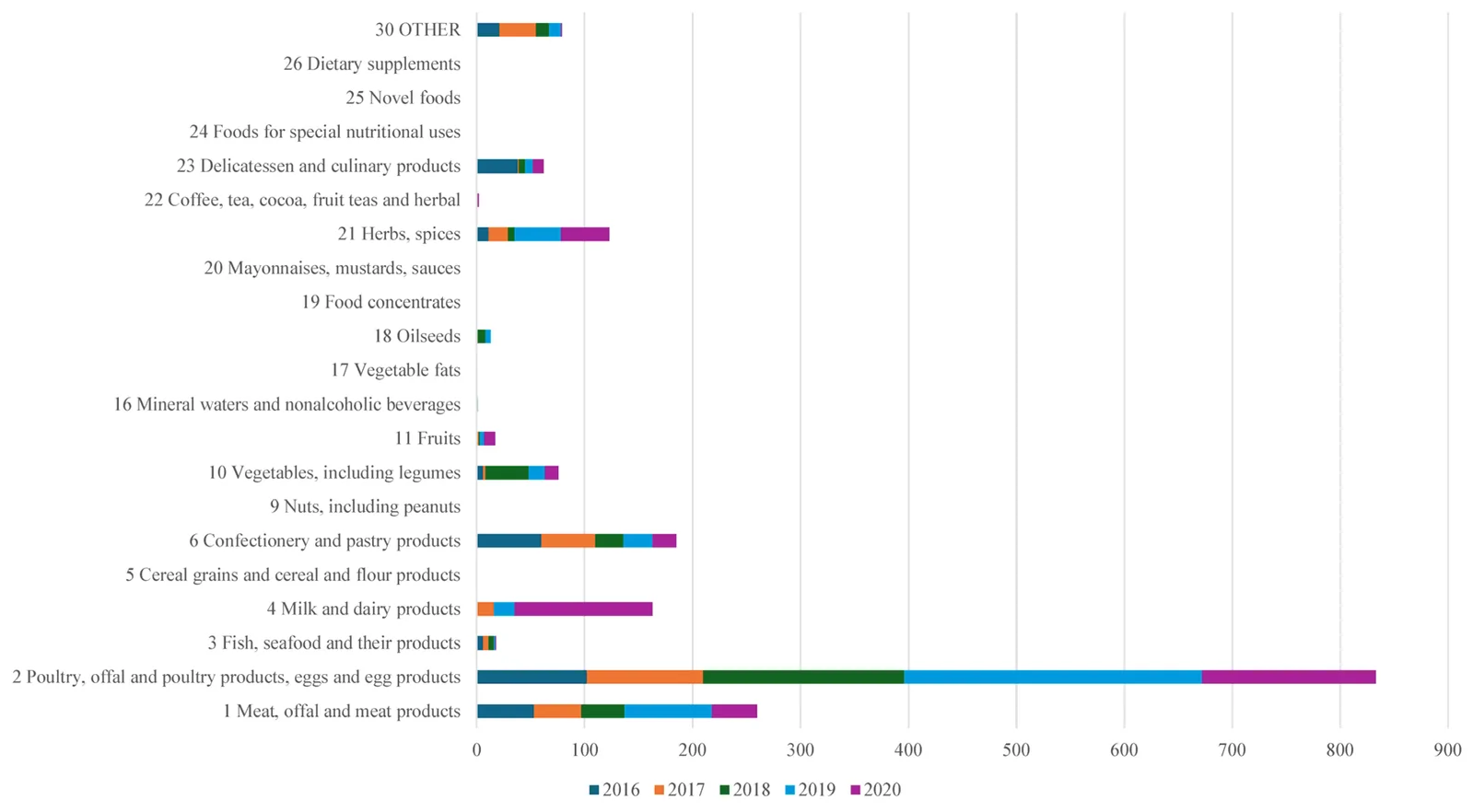
Disqualified samples by product groups (2016–2020).

A total of 380,314 samples were collected at the retail level between 2016 and 2020 (Figure 1), of which 1832 were disqualified due to failure to meet microbiological criteria or the presence of pathogens potentially posing a health risk (Figure 2).

Over a five-year period, the overall proportion of non-compliant samples among the samples tested for each microbiological criterion due to the detection of *Salmonella* spp., *L. monocytogenes*, and *Campylobacter* spp. or exceeding permissible limits for β-glucuronidase-positive *E. coli*, coagulase-positive staphylococci (*S. aureus*), or *L. monocytogenes* was as follows: *Campylobacter*—16.19% of tested samples; *Salmonella* spp.—0.66%; β-glucuronidase-positive *E. coli*—0.55%; coagulase-positive staphylococci (including *S. aureus*)—0.31%; *L. monocytogenes*—0.14%.

The proportion of disqualified samples differed significantly among the study years (Pearson’s chi-square test, χ^2^ = 162.69, df = 4, *p* < 0.001). The highest proportion of disqualified samples was observed in 2020 (0.734%; 95% CI: 0.669–0.806), whereas the lowest was observed in 2017 (0.324%; 95% CI: 0.288–0.364). It should be noted that the number of tested samples varied considerably between years, with fewer samples analysed in 2020 (n = 59,228) compared with previous years. Therefore, the observed increase in the proportion of disqualified samples should be interpreted considering the differences in annual sampling intensity.

As shown in Figure 2, the number of disqualified samples is minor compared to the number of samples tested (Figure 1). The prevalence of disqualified samples varied among food categories. The highest proportion was observed in poultry products (3.53%; 95% CI: 3.30–3.77), followed by other food categories (2.47%; 95% CI: 1.98–3.06) and meat products (1.03%; 95% CI: 0.91–1.16). Significant differences in the proportion of disqualified samples among food categories were observed (Pearson’s chi-square test with Monte Carlo’s simulation based on 10,000 replicates, χ^2^ = 5694.3, *p* < 0.001). Some groups had no disqualified samples during the entire period; they were “Cereal grains and cereal-flour products”, “Dietary supplements”, “Novel foods”, “Nuts, including peanuts”, “Vegetable fats”, “Food concentrates”, “Foodstuffs for special dietary purposes” and “Mayonnaise, mustard, sauces” groups. In several groups, an increase in the number of disqualified samples over time was observed. The trend analysis of selected food groups showed an increase in the percentage of disqualified samples in the “Poultry, offal and poultry products, eggs, and egg products” group. In 2018–2019, an increase in the percentage of disqualified samples was observed, followed by a decrease in 2020. The percentage of disqualified samples in this category in 2016–2020 was 4%, 2.1%, 4.3%, 4.5% (with a simultaneous increase in the number of samples), and 4.2% (with a simultaneous decrease in the number of samples), respectively. In the “Milk and dairy products” food category, no non-compliant samples were identified in 2016 and 2018. In 2017 and 2019, non-compliance was detected in 0.09% of the samples analyzed, while in 2020, this proportion increased to 1.01% in the “Herbs, spices” category of food products. In 2016–2018, the level of disqualification of products from this group was at a similar level, ranging from 0.2% to 0.4%. Since 2019, an increase in the percentage of disqualified samples has been observed to 0.9% in 2019 and 1.6% in 2020 (with a simultaneous decrease in the number of samples tested).

### 4.1. Salmonella *spp*.

Between 2016 and 2020, a total of 196,175 food samples were tested for the presence of *Salmonella* spp. The pathogen was detected in 1285 samples, corresponding to an overall prevalence of 0.66% (95% CI: 0.62–0.70%) (Table 1, Table 2, Table 3, Table 4 and Table 5). The annual prevalence of *Salmonella* spp. increased from 0.49% in 2016 and 0.47% in 2017 to 0.74% in 2018, 0.80% in 2019, and 0.89% in 2020. Differences among years were statistically significant (Pearson’s χ^2^ = 81.11, df = 4, *p* < 0.001). Logistic regression analysis further confirmed a significant temporal increase in the occurrence of *Salmonella* spp., with the odds of detecting the pathogen increasing by 18.6% annually (OR = 1.19, 95% CI: 1.14–1.23, *p* < 0.001).

The prevalence of *Salmonella* spp. differed significantly among product groups (Pearson’s χ^2^ = 3490.6, Monte Carlo’s simulation, *p* < 0.001). Logistic regression analysis demonstrated that the highest odds of *Salmonella* spp. detection were associated with “Poultry, offal and poultry products, eggs and egg products” (OR = 14.84, 95% CI: 12.26–17.97, *p* < 0.001). Significantly increased odds were also observed for “Other” (OR = 14.75, 95% CI: 10.88–19.99, *p* < 0.001), “Oilseeds” (OR = 7.25, 95% CI: 4.11–12.77, *p* < 0.001), “Meat, offal and meat products” (OR = 5.74, 95% CI: 4.58–7.19, *p* < 0.001), and “Herbs, spices” (OR = 4.32, 95% CI: 3.33–5.61, *p* < 0.001). Conversely, significantly lower odds of contamination were observed for “Fish, seafood and their products” (OR = 0.08, *p* = 0.002), “Delicatessen and culinary products” (OR = 0.40, *p* < 0.001), “Fruits” (OR = 0.46, *p* = 0.009), and “Milk and dairy products” (OR = 0.65, *p* = 0.007). The highest values were observed in “Poultry, offal and poultry products, eggs and egg products” (2.67% in 2016; 2.26% in 2017; 4.50% in 2018; 4.28% in 2019; and 4.32% in 2020). A moderate increase was also recorded in “Meat, offal and meat products” (from 1.34% to 1.85%). In “Milk and dairy products”, non-compliance with the criteria reached 0.95% in 2020, which is significant given the simultaneous reduction in the number of samples tested. In the “Fruits” group, the share of disqualified samples increased gradually from 0.00% in 2016 to 0.29% in 2020. Although the overall level remained low, the upward trend was most apparent in 2019–2020. In “Vegetables, including legumes”, the percentage of disqualified samples was as follows: 0.04% (2016), 0.08% (2017), 0.58% (2018), 0.04% (2019), 0.52% (2020). In the category “Fish, seafood and their products”, non-compliance with the contamination criteria was detected only in 2016 (0.06%). In subsequent years, no presence of *Salmonella* spp. was found. For “Confectionery and pastry products”, small and irregular fluctuations were noted, without a clear upward tendency. In the “Oilseeds” category, no disqualified samples were recorded in 2016. No data were available for this category in 2017. The first detection of *Salmonella* spp. occurred in 2018, with a prevalence of 4.02%, followed by a decrease in 2019 to 1.64%. In 2020, no disqualified samples were identified. In the “Herbs, spices” category, contamination levels were low but present throughout the study period. The prevalence was 0.49% in 2016, increased to 0.81% in 2017, then declined to 0.41% in 2018. In subsequent years, a marked increase was observed, reaching 1.87% in 2019, followed by a slight decrease to 1.77% in 2020. For “Coffee, tea, cocoa, fruit teas and herbal” products, no contamination was reported between 2016 and 2019, while in 2020, 1.67% was recorded. “Delicatessen and culinary products” showed relatively low and declining levels: 0.44% in 2016, 0% in both 2017 and 2018, 0.03% in 2019, and again 0% in 2020, indicating decreasing relevance. The group classified as “Other products” exhibited the highest variability in prevalence across the study period. The disqualification rate was 3.11% in 2016, followed by a pronounced increase to 8.37% in 2017. In 2018, a substantial decline was observed, with prevalence decreasing to 1.12%. This was followed by a moderate increase to 2.72% in 2019, after which a marked reduction occurred, reaching 0.41% in 2020.

### 4.2. L. monocytogenes

Between 2016 and 2020, a total of 150,444 food samples were tested for the presence of *Listeria monocytogenes*. The pathogen was detected in 207 samples, corresponding to an overall prevalence of 0.138% (95% CI: 0.120–0.158%) (Table 1, Table 2, Table 3, Table 4 and Table 5). The proportion of disqualified samples differed significantly between the study years (χ^2^ = 142.78, df = 4, *p* < 0.001). The overall proportion of non-compliant samples in the first four years of the analysis fluctuated between 0.05% and 0.12%, whereas in 2020 a marked increase to 0.39% was recorded. The lowest prevalence was observed in 2017 (0.054%; 95% CI: 0.034–0.085%), whereas the highest prevalence occurred in 2020 (0.386%; 95% CI: 0.316–0.470%). In the remaining years, prevalence ranged from 0.071% in 2019 to 0.120% in 2018, while in 2016 it reached 0.117%. Logistic regression demonstrated a significant increasing temporal trend in the occurrence of *L. monocytogenes*. The odds of detecting the pathogen increased by approximately 43.5% per year (OR = 1.435, 95% CI: 1.294–1.591, *p* < 0.001), indicating a systematic increase in contamination during the study period.

A more detailed analysis revealed considerable variation in the prevalence of *L. monocytogenes* among individual food categories over the study period. In the early years, the highest proportion of non-compliant samples was observed in “Delicatessen and culinary products”, reaching 0.84% in 2016. In 2018, the highest prevalence was recorded in “Vegetables, including legumes” (0.83%), followed by “Fish, seafood and their products” (0.45%). In 2020, the highest prevalence was observed in “Herbs, spices” (1.43%) and “Milk and dairy products” (1.05%). Detection of *L. monocytogenes* in the latter category was mainly associated with samples from the “Desserts and ice creams to be eaten with a spoon” subgroup exceeding the microbiological criterion. Overall, the prevalence of *L. monocytogenes* differed significantly among food categories (Pearson’s χ^2^ = 105.02, *p* = 0.015), indicating that the type of food product significantly influenced the probability of pathogen detection. Logistic regression analysis further demonstrated significantly higher odds of contamination for several product groups compared with the reference category. The highest odds were observed for “Delicatessen and culinary products” (OR = 18.00, 95% CI: 3.53–91.70, *p* < 0.001), followed by “Vegetables, including legumes” (OR = 15.19, 95% CI: 2.96–78.02, *p* = 0.001), “Fish, seafood and their products” (OR = 14.14, 95% CI: 2.67–74.95, *p* = 0.002), “Herbs, spices” (OR = 12.48, 95% CI: 2.38–65.50, *p* = 0.003), “Milk and dairy products” (OR = 9.45, 95% CI: 1.87–47.75, *p* = 0.007), and “Confectionery and pastry products” (OR = 5.56, 95% CI: 1.08–28.62, *p* = 0.040). No statistically significant increase in the odds of contamination was observed for the remaining food categories (*p* > 0.05). Although the overall occurrence of *L. monocytogenes* increased significantly during the study period (OR per year = 1.44, 95% CI: 1.29–1.59, *p* < 0.001), temporal patterns differed between individual food categories. “Confectionery and pastry products” showed a gradual decrease in the proportion of non-compliant samples from 0.15–0.17% in 2016–2017 to 0% in 2020. Likewise, following the peak observed in 2018, the prevalence in “Vegetables, including legumes” and “Fish, seafood and their products” declined in subsequent years. In contrast, the highest prevalence in “Herbs, spices” and “Milk and dairy products” was recorded in 2020, indicating that changes over time were not uniform across food categories. It should also be noted that certain categories consistently demonstrated a high level of compliance, with no disqualified samples recorded throughout the study period. This particularly applies to groups such as “Fruits”, “Cereal grains and cereal and flour products”, “Nuts, including peanuts”, “Foods for special nutritional uses”, “Dietary supplements”, “Novel foods”, and “Coffee, tea, cocoa, fruit teas and herbal”.

### 4.3. E. coli (Including STEC)

Over the years 2016–2020, the proportion of disqualified samples in different food product groups remained low; a total of 22,695 food samples were analysed, of which 125 (0.55%; 95% CI: 0.46–0.66%) were disqualified due to non-compliance with the criterion for *E. coli.* The proportion of disqualified samples differed significantly across the study years. The lowest proportion was observed in 2017 (0.29%; 95% CI: 0.18–0.46%), whereas the highest was recorded in 2020 (1.36%; 95% CI: 0.98–1.90%). In the remaining years, the proportions of disqualified samples were 0.39% in 2016, 0.59% in 2018, and 0.56% in 2019.

Pearson’s chi-squared test demonstrated significant differences in the frequency of sample disqualification between years (χ^2^ = 39.84, df = 4, *p* < 0.001) (Table 1, Table 2, Table 3, Table 4 and Table 5). Significant differences were also observed among product groups (Pearson’s chi-squared test with Monte Carlo’s simulation: χ^2^ = 728.2, *p* < 0.001). The highest proportion of disqualified samples was observed in “Poultry, poultry products, eggs, and egg products” (37.5%); however, this estimate was based on a limited number of samples (n = 16) and should therefore be interpreted with caution. Among the more extensively sampled product categories, the highest proportion of disqualified samples was found in “Confectionery and pastry products” (8.16%), followed by “Milk and dairy products” (1.86%) and “Meat products” (1.65%). The lowest proportions were observed in “Mineral waters and nonalcoholic beverages” (0.015%), “Fruits” (0.16%), and “Vegetables” (0.26%).

Using “Meat and meat products” as the reference category, logistic regression analysis showed that “Confectionery and pastry products” had more than fivefold higher odds of sample disqualification (OR = 5.42; 95% CI: 2.83–10.42; *p* < 0.001). In contrast, significantly lower odds of disqualification were observed for “Fruits” (OR = 0.10; 95% CI: 0.04–0.25; *p* < 0.001), “Vegetables” (OR = 0.16; 95% CI: 0.09–0.27; *p* < 0.001), and “Mineral waters and nonalcoholic beverages” (OR = 0.01; 95% CI: 0.003–0.067; *p* < 0.001). No significant differences were found between “Meat and meat products” and “Milk and dairy products” (OR = 1.13; *p* = 0.592), “Delicatessen and culinary products” (OR = 0.89; *p* = 0.857), or the category classified as “Other” (OR = 0.30; *p* = 0.148) A year-specific analysis revealed considerable variation in the distribution of disqualified samples among product groups. In 2016, the highest proportion of disqualified samples was observed in “Meat, offal and meat products” (2.34%) and “Other” (1.11%). In 2017, the highest proportions occurred in “Milk and dairy products” (3.18%) and “Delicatessen and culinary products” (2%). In 2018, the largest share of non-compliant samples was also recorded in “Meat, offal and meat products” (1.94%). Elevated proportions were again observed in 2019 in the “Delicatessen and culinary products” (3.85%) and “Milk and dairy products” (3.11%) categories”. In 2020, a marked increase in disqualified samples was recorded, particularly in “Poultry, offal and poultry products, eggs and egg products” (60%), “Confectionery and pastry products” (48%), “Milk and dairy products” (8.33%), and “Fruits” (1.82%).

However, the high proportion of disqualified samples in the “Poultry, offal and poultry products, eggs and egg products” and “Confectionery and pastry products” categories should be interpreted with caution because they were based on relatively small numbers of analysed samples (10 and 25 samples, respectively), which were collected within the framework of targeted consumer complaint investigations. Some product groups consistently showed no disqualified samples throughout the entire study period. This applies to “Fish, seafood and their products”, “Cereal grains and cereal and flour products”, “Herbs, spices”, “Foods for special nutritional uses”, and “Dietary supplements”. These groups can be regarded as the most stable in terms of quality during the period analyzed, with no disqualified samples. Overall, the proportion of disqualified samples increased from 0.39% in 2016 to 1.36% in 2020. This increase was consistent with the significant temporal trend identified by the logistic regression analysis (OR = 1.42; 95% CI: 1.23–1.63; *p* < 0.001), indicating an increasing probability of sample non-compliance over the study period. The largest increase occurred in the final year of observation and was driven by the high proportion of non-compliant samples in the “Poultry, offal and poultry products, eggs and egg products” group. By contrast, groups such as “Fish, seafood and their products”, “Herbs, spices”, “Foods for special nutritional uses”, and “Dietary supplements” remained the most stable.

### 4.4. Coagulase-Positive Staphylococci (Including S. aureus)

Analysis of data on the presence of coagulase-positive staphylococci (including *S. aureus*) in food samples between 2016 and 2020 indicated that overall levels in most product groups were low. Of 10,391 food samples examined for the presence of staphylococci, 32 samples tested positive, corresponding to an overall prevalence of 0.31% (95% CI: 0.22–0.44%) (Table 1, Table 2, Table 3, Table 4 and Table 5). The annual proportion of disqualified samples varied throughout the study period, ranging from 0% in 2017 to 0.61% in 2018 (95% CI: 0.33–1.12%). In 2016, the prevalence was 0.31% (95% CI: 0.15–0.65%), followed by 0.20% (95% CI: 0.09–0.47%) in 2019 and 0.57% (95% CI: 0.31–1.05%) in 2020. Although the estimated odds of detecting coagulase-positive staphylococci (including *S. aureus*) increased by 23.4% per year (OR = 1.234; 95% CI: 0.959–1.589), this temporal trend was not statistically significant (*p* = 0.102). Nevertheless, the distribution of positive samples differed significantly between years (Pearson’s χ^2^ = 16.72, *p* = 0.002).

Many samples showed no disqualification, indicating a high level of microbiological safety of the monitored products during the study period. Nevertheless, several exceptions were observed. No positive samples were detected in “Poultry, offal and poultry products, eggs and egg products”, “Fish, seafood and their products”, “Meat, offal and meat products”, “Vegetables, including legumes”, or most of the remaining product categories. Marked differences were also observed among product groups (Pearson’s χ^2^ = 171.46, *p* = 0.00). The highest prevalence was found in “Confectionery and pastry products”, where 5 of 89 samples were disqualified (5.62%, 95% CI: 2.42–12.50%). Elevated prevalence was also observed in “Other” (2.24%, 95% CI: 1.34–3.73%) and “Delicatessen and culinary products” (0.53%, 95% CI: 0.18–1.55%). In contrast, “Milk and dairy products”, despite accounting for the largest number of tested samples (7139), showed a relatively low overall prevalence of 0.14% (95% CI: 0.08–0.26%). A more detailed analysis by year showed that most product categories exhibited no disqualified samples, indicating a generally high level of microbiological safety throughout the study period. The most pronounced non-compliance was observed in “Confectionery and pastry products” in 2019, where 5 of 40 samples were disqualified (12.50%). Relatively high proportions of disqualified samples were also recorded in the “Other” category, reaching 9.00% in 2018 and 1.85% in 2016. In 2020, “Milk and dairy products” accounted for 0.79% of samples, representing the largest number of positive samples detected within a single product group during the study period. In “Delicatessen and culinary products”, contamination was identified in 2016 (0.95%) and 2018 (0.94%). Logistic regression analysis confirmed significant differences in the odds of contamination between product groups. Compared with the reference category, samples classified as “Other” had the highest odds of coagulase-positive staphylococci (including *S. aureus*) (OR = 16.13, 95% CI: 7.26–35.85; *p* < 0.001), followed by “Delicatessen and culinary products” (OR = 4.23, 95% CI: 1.26–14.22; *p* = 0.020). No statistically significant differences were identified for “Poultry, offal and poultry products, eggs and egg products”, “Fish, seafood and their products”, or “Novel foods” (*p* > 0.05). Overall, contamination with coagulase-positive staphylococci (including *S. aureus*) was infrequent during the study period. Although significant differences were observed between years and product categories, the occurrence of positive samples was sporadic and did not indicate a persistent upward trend. Instead, contamination was concentrated in a limited number of product groups, particularly “Confectionery and pastry products”, “Other”, and, to a lesser extent, “Delicatessen and culinary products”, suggesting that these categories warrant continued targeted microbiological surveillance.

### 4.5. Campylobacter *spp*.

Based on the analysis of data concerning the presence of *Campylobacter* spp. in various groups of food products, a significant variation in the percentage of disqualified samples was observed (Table 1, Table 2, Table 3, Table 4 and Table 5). Between 2016 and 2020, a total of 1056 food samples were tested for the presence of *Campylobacter* spp. The pathogen was detected in 171 samples, corresponding to an overall prevalence of 16.19% (95% CI: 14.09–18.54%). The proportion of disqualified samples varied significantly over the study period (Pearson’s χ^2^ = 27.70, *p* < 0.001). The lowest prevalence was observed in 2016, when *Campylobacter* was detected in 15.5% of samples (95% CI: 9.27–24.70%). The prevalence subsequently decreased slightly to 10.6% in 2017 (95% CI: 7.21–15.20%), before increasing to 17.3% in 2018 (95% CI: 12.50–23.40%). The highest prevalence was recorded in both 2019 and 2020, when 22.1% of samples were positive (66/299 and 29/131, respectively). Logistic regression demonstrated a small but statistically significant increasing temporal trend, with the odds of detecting *Campylobacter* increasing by approximately 0.1% per year (OR = 1.001, 95% CI: 1.000–1.001; *p* = 0.000819). Marked differences were also observed between product groups (Pearson’s χ^2^ = 76.66, *p* < 0.001). The highest prevalence of *Campylobacter* was found in “Poultry, offal and poultry products, eggs and egg products”, where 148 of 594 samples (24.9%; 95% CI: 21.6–28.5%) were disqualified. In contrast, “Meat, offal and meat products” showed a substantially lower prevalence of 5.3% (23/434; 95% CI: 3.56–7.83%). No positive samples were detected in “Confectionery and pastry products”, “Fish, seafood and their products”, “Foods for special nutritional uses”, or “Other”. However, these categories were represented by very small numbers of samples and therefore do not allow reliable comparisons.

Compared with the reference category, “Poultry, offal and poultry products, eggs and egg products”, samples from “Meat, offal and meat products” had approximately 83% lower odds of *Campylobacter* contamination (OR = 0.172, 95% CI: 0.109–0.271, *p* < 0.001), confirming poultry products as the principal reservoir of *Campylobacter* among the food categories examined.

## 5. Discussion

Food safety is a key element of public health protection in the European Union. Monitoring the presence of pathogenic bacteria in food products aims to support food quality control, identification of new threats in the food safety sector, and minimizing the risk of foodborne poisoning and infections [15,16]. Applicable legal regulations, including Commission Regulation (EC) No. 2073/2005 on microbiological criteria for foodstuffs, provide the basis for assessing the microbiological quality of food in Member States. Analysis of information on result the samples tested allows us to track trends and changes that have occurred in recent years regarding known and new microbiological threats in food at the retail stage. However, this surveillance approach has certain limitations. As mentioned above, the data reflect only the retail stage of the food chain, and the number of samples collected varied among different food categories. Moreover, the results do not allow estimation of the actual impact of the detected microbiological hazards on the occurrence of foodborne illnesses.

Between 2016 and 2020, 380,314 food samples were tested at the retail level in Poland. Of these, 1832 (0.48%) did not meet the requirements specified in both the regulation and national risk analysis guidelines. The non-compliances mainly concerned the presence of *Campylobacter* spp., *Salmonella* spp., *L. monocytogenes*, *E. coli*, including STEC and coagulase-positive staphylococci, including *S. aureus*. These microorganisms have been a major threat to food safety for years [16,17,18,19,20]. In the years 2016–2020 in the European Union, these microorganisms were the most common cause of bacterial food poisoning and were responsible for causing: campylobacteriosis (853,604 cases), salmonellosis (379,278 cases), STEC/VTEC infection (27,215 cases) and listeriosis (11,343 cases) [21].

To provide a broader epidemiological context for the public-health relevance of the pathogens included in the present study, national data on reported human infections were also considered for the period 2016–2020 [22,23,24,25,26]. These epidemiological data should, however, be interpreted independently from the food surveillance results presented in this study, as trends in pathogen detection in food products cannot be directly translated into corresponding changes in the incidence of human infections. In Poland, salmonellosis was the most frequently reported infection throughout the analysed period. Between 2016 and 2018, the number of cases remained relatively stable, ranging from 9662 to 9709 (25.15–25.27 per 100,000 population), before decreasing to 8928 cases (23.24) in 2019 and further to 5092 cases (13.27) in 2020. A similar downward trend was observed for listeriosis, although the overall number of reported cases was considerably lower. Between 2017 and 2019, the number of cases remained relatively stable at 117–124 (0.30–0.32), whereas in 2020 it decreased markedly to 51 cases (0.13). Diarrhoeagenic *E. coli* infections also showed relatively stable notification rates before 2020. Between 2016 and 2019, the number of reported cases ranged from 274 to 332 (0.71–0.86), followed by a substantial decline to 72 cases (0.19) in 2020. A comparable pattern was found for campylobacteriosis, although some fluctuations were observed during the study period. The number of cases increased from 790 (2.06) in 2016 to 885 (2.30) in 2017, before declining to 738 (1.92) in 2018, 720 (1.87) in 2019, and 443 (1.15) in 2020. In contrast, staphylococcal food poisoning initially showed an increasing trend, followed by a pronounced decline. The number of reported cases increased from 38 (0.10) in 2016 to 51 (0.13) in 2017 and 68 (0.18) in 2018, before decreasing to 15 (0.04) in 2019 and only 4 (0.01) in 2020.

The analysed period (2016–2020) also included the first year of the COVID-19 pandemic. Based on the analysis of the study results, no clear impact of the pandemic on the proportion of non-compliant samples was observed. However, it should be noted that the number of collected samples in 2020 was lower for most food categories compared with previous years, which was related to limitations in both sample collection and laboratory testing capacity during this period.

### 5.1. Salmonella *spp*.

The results of studies conducted between 2016 and 2020 indicate an overall increase in the occurrence of *Salmonella* spp. in the food samples, with substantial differences among product groups. Although overall prevalence remained relatively low, the temporal increase and marked variation between food categories indicate that the risk of *Salmonella* spp. contamination is strongly dependent on the type of product. The highest values were detected in poultry, offal and poultry products, eggs and egg products, confirming the importance of poultry and eggs as major vehicles of *Salmonella* spp. Similar observations have been reported in European and national studies, where poultry and eggs consistently remain the most common source of salmonellosis in humans [15,16,17,18,19,20,27]. *S. enteritidis* remains the dominant serotype in Poland, which is particularly often associated with eggs and poultry products. The increase in this category may be related to the continued presence of certain serotypes in production flocks. The increased occurrence observed in meat, offal and meat products supports the continued importance of microbiological surveillance of meat products [28]. Although milk and dairy products generally showed relatively low odds of *Salmonella* spp. detection, the increase observed in the final year of the study deserves attention, particularly given the simultaneous reduction in the number of samples tested. This finding should therefore be interpreted cautiously. This is likely due to insufficient heat treatment during processing or secondary contamination at later stages, such as packaging and storage. Similar problems have been reported in other EU countries, where dairy products have been sporadically associated with salmonellosis outbreaks, despite strict hygiene standards [17]. “Herbs, spices” also represented a relevant category, with higher contamination levels observed towards the end of the study period. This pattern is consistent with previous reports highlighting herbs and spices as an important source of contamination. Their vulnerability is related to long storage times, low water activity, and the absence of heat treatment before consumption. In addition, contamination is often linked to the drying process and to imports from regions with lower hygiene standards [29,30].

The detection of *Salmonella* spp. in other dry products, including coffee, tea, cocoa, fruit teas and herbal products, as well as oilseeds [31,32]. Further highlights the ability of this pathogen to persist in low-water-activity environments. Although contamination in these categories was irregular and did not indicate a consistent temporal trend, its occurrence is relevant because *Salmonella* spp. may survive for prolonged periods in dry products. Similar observations have been reported for nuts and seeds, which have previously been implicated in foodborne outbreaks [16,17,32]. The occurrences of *Salmonella* spp. in “Fruits” and “Vegetables, including legumes” were generally low and variable. Nevertheless, its detection in these products confirms that foods of plant origin may also act as vehicles for transmission. Previous EFSA reports [15,16] and studies by Lee et al. [33] and Canning et al. [34] have similarly highlighted the potential role of fruits in *Salmonella* spp. infections. In vegetables, contamination may be influenced by environmental and technological factors, including fertilization, irrigation, harvesting conditions, and processing practices. Fresh leafy vegetables may be of particular concern when consumed as ready-to-eat products [35]. In contrast, a steady decline in the incidence of “Delicatessen and culinary products” has been demonstrated, suggesting the effectiveness of the hygiene procedures and control systems in place [15,16]. *Salmonella* spp. have been detected only incidentally in “Fish, seafood and their products”, consistent with the observations of Porto et al. [36], indicating a marginal role for this group in *Salmonella* spp. transmission.

The results indicate that the control of *Salmonella* spp. should extend beyond traditionally high-risk animal products, such as poultry, meat, and eggs, to include plant products, spices, and herbs. Addressing this issue requires consistent application of strict hygiene standards throughout the supply chain and careful monitoring of imported raw materials.

### 5.2. L. monocytogenes

The overall level of non-compliance in our studies was very low (0.05–0.12% of samples in 2016–2019, rising to 0.39% in 2020), which is consistent with EU surveillance data. EFSA and ECDC reports show that most of the ready-to-eat foods comply with the microbiological criterion of 100 CFU/g for *L. monocytogenes*. Across most ready-to-eat food categories, the proportion of non-compliant samples is generally below 1%, although higher values have occasionally been reported for specific product categories, particularly ready-to-eat fishery products” [16,17,18,19,20,21]. According to EFSA and ECDC, ready-to-eat fish, dairy and meat products are among the food categories most frequently associated with *L. monocytogenes* contamination and listeriosis. Consistent with this observation, analysis of the RASFF database showed that since 2008, the largest proportion of *Listeria* notifications has involved fish and fish products (42%), followed by milk and milk products (27%), and meat products (18%) [19]. In our study, non-compliance was most frequently identified in milk and dairy products and delicatessen and culinary products, while cases were also recorded in vegetables, confectionery and pastry products, herbs and spices, fish and seafood products, and, less frequently, poultry and meat products. This distribution generally supports the importance of product type in the occurrence of *L. monocytogenes*, although the patterns observed in our study did not fully correspond to those reported in European surveillance data. FSA states that at the retail stage, non-compliance levels rarely exceed 1%, and in the milk, RTE category, they were <0.1% throughout all years [19]. For comparison, a meta-analysis showed the presence of *L. monocytogenes* at a level of approximately 4.6% in milk and dairy products and approximately 8.7% in the entire milk production chain [37]. In turn, in raw fish from Poland, the presence of *Listeria* spp. was found in 18.9% of samples [38]. Although herbs and spices are not the main source of listeriosis, they may contain these pathogens. A study by the FSAI (Ireland) showed that herbs and spices are generally safe—*L. monocytogenes* was not detected in any of the 841 dried products tested [39]. Our finding of a relatively high percentage of non-compliance in “Herbs, spices” in 2020 (1.43%) is unusual and may indicate a product-specific or temporary contamination problem that warrants further attention. This result highlights the need for monitoring in such raw materials as well, especially since storage conditions and low levels of processing can sometimes allow bacteria to survive. The literature also indicates the seasonal presence of pathogens such as *L. monocytogenes* on raw vegetables and herbs—*L. monocytogenes* were detected in 0.6–9.1% of vegetable samples and up to approximately 10% of frozen vegetables in other studies [40,41]. Compared to these reports, our generally lower than those reported in the literature [33,38]. Overall, despite the low level of non-compliance, the observed increase over the study period indicates that the occurrence of *L. monocytogenes* was not stable over time. Moreover, temporal patterns differed between food categories, suggesting that the overall increase was not uniformly distributed across all types of food. These differences support continued monitoring of *L. monocytogenes*, with particular attention to product groups in which increases in non-compliance were observed, especially herbs and spices and milk and dairy products.

### 5.3. E. coli (Including STEC)

The results indicate that overall, the percentage of samples non-compliant with microbiological requirements for *E. coli* (including STEC) was low. EU surveillance data indicate low and variable detection of STEC in food samples in 2019 and 2020, depending on the food matrix [15,16]. Our data showed that the proportion of non-compliant samples ranged from 0.39% in 2016 to 1.36% in 2020. Nevertheless, the increase observed over the study period and the differences between product groups indicate that non-compliance was not uniformly distributed over time or across food categories. Some product groups showed substantially higher levels of non-compliance, although these findings should be interpreted in relation to the number and type of samples examined. In 2016, meat, offal and meat products (2.34%) constituted the category with the highest percentage of non-compliant samples. These results are consistent with EFSA and ECDC reports, which identify beef and beef products as among the main sources of STEC in Europe. The 2019 One Health report indicates that approximately 4.1% of meat and meat product samples in EU countries were positive for STEC [15]. Higher isolation rates were reported in a study conducted in Italy in 2017, where STEC was isolated from 8.4% of fresh beef samples [42]. Milk and dairy products showed a variable but notable proportion of non-compliant samples, amounting to 3.18% in 2017, 3.11% in 2019, and increasing to 8.33% in 2020. At the same time, this trend occurred alongside a reduction in the average annual number of samples in this category by more than 80%. These results correspond to the EFSA report, according to which approximately 0.9% of cheese samples in the EU were positive for STEC [15]. An important finding concerned confectionery and pastry products, which emerged as one of the product groups with a comparatively high level of non-compliance in the overall analysis. The particularly high proportion observed in 2020 should, however, be interpreted cautiously because it was based on a small number of samples collected as part of targeted consumer complaint investigations. This sampling context may have increased the probability of detecting non-compliant products and limits direct comparison with categories examined through broader routine surveillance.

*E. coli* is also frequently detected in fruits and vegetables. In 2018, the highest proportion of disqualified samples was observed in "vegetables, including legumes (0.92%), while in 2020, this applied to fruits (1.82%). Although these values were lower than those recorded for products of animal origin, numerous studies confirm that plants can also serve as a vehicle for *E. coli*, especially for STEC transmission [43,44]. According to EFSA, the overall proportion of STEC-positive vegetable samples in the EU remains below 0.2% [17]. However, even single contamination events may have far-reaching public health consequences. A notable example is the large outbreak of STEC O104:H4 infections in Germany in 2011, which was traced back to contaminated sprouts [45] or an outbreak of Shiga toxin-producing *Escherichia coli* (STEC) O157:H7 associated with contaminated lettuce [46]. These findings highlight that, while the probability of contamination of vegetables and fruits is relatively low, the potential epidemiological impact of such events can be substantial. RTE products, such as delicatessen and culinary products, showed periodically elevated rates of non-compliant samples. These results are consistent with the literature, which emphasizes that RTE foods may pose a particular risk because they are not subject to further heat treatment. The EFSA BIOHAZ report [47] highlights the need for systematic monitoring of this product group. In contrast, no non-compliant samples were observed throughout the study period in several product groups, including fish, seafood and their products; cereal grains and cereal and flour products; herbs, spices; foods for special nutritional uses; and dietary supplements. This consistent absence of non-compliant samples indicates a high level of compliance in these categories during the period analyzed, although it should not be interpreted as evidence of an absence of microbiological risk. The findings indicate a low level of non-compliance with the *E. coli* criterion, but also an increasing temporal pattern and marked heterogeneity between food categories. The pronounced increases observed in selected categories in 2020 should be interpreted cautiously, particularly where the number of analyzed samples was small, and sampling was targeted rather than representative of routine surveillance. These observations underline the importance of considering both product type and sampling strategy when interpreting changes in microbiological non-compliance over time.

### 5.4. Coagulase-Positive Staphylococci (S. aureus)

In the present study, non-compliance due to coagulase-positive staphylococci, including *S. aureus*, was generally uncommon but differed between product categories, with the highest prevalence observed in confectionery and pastry products. Previous studies have also reported microbiological contamination of creamy confectionery products [48]. Such foods may be particularly susceptible to contamination during preparation and handling because human skin and mucous membranes are important reservoirs of staphylococci. The risk may be increased in ready-to-eat products subjected to extensive manual handling without subsequent heat treatment. In contrast, milk and dairy products showed a low level of non-compliance despite being the most extensively examined category. Zhang et al. [49] reported a substantially higher prevalence of staphylococci in raw milk than in dairy products and pasteurized milk. However, direct comparison is limited because the present data concern regulatory non-compliance across a broad category of milk and dairy products rather than the prevalence of staphylococci specifically in raw milk. Despite the generally low level of non-compliance, coagulase-positive staphylococci remain relevant to food safety because certain strains can produce heat-stable enterotoxins that may retain biological activity after heat treatment. Staphylococcal food poisoning has been associated with various foods, including confectionery, dairy, meat and fish products, salads, vegetable products, and ice cream [50].

Overall, the findings indicate a high level of microbiological compliance, with non-compliance concentrated in selected food categories rather than showing a persistent increase over time. Continued risk-based surveillance should therefore focus particularly on ready-to-eat foods susceptible to contamination during preparation and handling.

### 5.5. Campylobacter *spp*.

According to EFSA and ECDC reports, EU countries tested samples against the *Campylobacter* spp. process hygiene criterion specified in Regulation (EC) No. 2073/2005 between 2016 and 2020, particularly from neck skin samples. Of the reported results between 2016 and 2020, 27.6% (17,695 out of 66,510 samples) were positive for *Campylobacter* spp. in the fresh meat category [16,17,18,19,20]. In our study, we analyzed data from monitoring based on the detection of *Campylobacter* spp. in samples taken from the market. Overall, *Campylobacter* spp. were detected in 16.19% (171/1056) of all food samples examined. Although this prevalence was lower than that reported in EU monitoring, direct comparisons should be interpreted with caution because of differences in sampling strategies, food matrices, and stages of the food chain. According to data from EFSA and ECDC, the rejection rate for the categories fresh meat from broilers and fresh meat from turkeys was 32%, corresponding to 14,402 out of 44,607 samples tested. In our study, the highest prevalence was observed in the category poultry, offal and poultry products, eggs and egg products, in which 24.9% (148/594) of samples were positive for *Campylobacter* spp. This proportion was lower than that reported for fresh broiler and turkey meat in EU monitoring; however, the broader composition of the product category examined in the present study should be considered when interpreting this comparison.

A survey conducted in 2011 as part of the Food Safety Authority of Ireland’s national microbiological surveillance program on raw chicken at retail revealed that *Campylobacter* spp. were detected in 50.2% of samples using a quantitative method, with 5.9% of samples contaminated at levels exceeding 1000 colony-forming units per gram (CFU/g) [51]. In the Zbrun et al. [52] study, retail chickens showed the highest percentage of *Campylobacter*-positive samples, at 83.3% (25 of 30 samples). Quantitative analysis showed that contamination levels in poultry carcasses ranged from 0 to 3.71 log10 CFU per carcass. While in the group “Meat, offal and meat products”, the average value was 5%, which indicates a smaller presence of the pathogen; this compares to 15% (3293 out of 21,903) of sample disqualifications in this category reported in European Union Member States. In beef samples available on the Italian market, *Campylobacter* spp. were detected in only 0.58% of samples. Only one beef sample had a contamination level exceeding 10 CFU/g (240 CFU/g). In contrast, among the Campylobacter-positive poultry meat samples, 48.15% had contamination levels ≥ 10 CFU/g, whereas the remaining 51.85% contained fewer than 10 CFU/g [53]. These observations further support the markedly greater occurrence of *Campylobacter* in poultry than in other meat products, although differences in study design, analytical methods, and food matrices should be considered when comparing prevalence estimates between studies. A significant variation in the proportion of *Campylobacter* spp. positive samples was also observed over the study period. The data indicate an increase in the proportion of positive samples during the latter part of the study period. Nevertheless, this temporal pattern should be interpreted cautiously because annual differences may also reflect changes in the number and composition of samples tested. In summary, the results indicate that poultry products represented the food category with the highest prevalence of *Campylobacter* spp. among the categories examined, whereas substantially lower prevalence was observed in meat, offal and meat products. These findings support the importance of continued microbiological monitoring and control measures targeting poultry products. At the same time, the absence of positive results in several less frequently sampled food categories should not be interpreted as evidence of negligible risk because of the limited number of samples examined.

## 6. Conclusions

Many microbiological threats have long been recognized, and current legislation includes procedures and guidelines related to the control of the highest-risk foods. However, the food market is extremely dynamic, and we often encounter new products such as novel foods. All these factors force food safety authorities to take a critical approach to monitoring the food market and to constantly keep up with the latest knowledge. Monitoring the presence of pathogens in food is essential to protecting public health and ensuring food safety. Regular inspections detect contamination and minimize the risk of infection in consumers. Quantitative, qualitative, and molecular analyses enable risk assessment and identification of pathogen sources, supporting effective prevention strategies. Systematic surveillance helps reduce the incidence of foodborne illnesses. Implementing such measures is crucial to reducing the number of poisonings and potentially outbreaks caused by pathogenic microorganisms.

## Figures and Tables

**Table 1:** Detailed Results of Monitoring and Official Control Studies Conducted on the Retail Market in Poland in 2016.

	2016														
	*Salmonella* spp.			*L. monocytogenes*			*E. coli*			Coagulase-Positive Staphylococci (Including *S. aureus*)			*Campylobacter* spp.		
PRODUCT GROUP	Tested Samples	Disqualified Samples	Percent of Disqualified Samples	Tested Samples	Disqualified Samples	Percent of Disqualified Samples	Tested Samples	Disqualified Samples	Percent of Disqualified Samples	Tested Samples	Disqualified Samples	Percent of Disqualified Samples	Tested Samples	Disqualified Samples	Percent of Disqualified Samples
1 MEAT, OFFAL AND MEAT PRODUCTS	2688	36	1.34%	1989	0	0.00%	427	10	2.34%	3	0	0.00%	130	7	5.38%
2 POULTRY, OFFAL AND POULTRY PRODUCTS, EGGS AND EGG PRODUCTS	3329	89	2.67%	565	0	0.00%	-	-	-	266	0	0.00%	64	13	20.31%
3 FISH, SEAFOOD AND THEIR PRODUCTS	1608	1	0.06%	1586	5	0.32%	5	0	0.00%	6	0	0.00%	-	-	-
4 MILK AND DAIRY PRODUCTS	8076	0	0.00%	8456	0	0.00%	500	0	0.00%	1425	0	0.00%	-	-	-
5 CEREAL GRAINS AND CEREAL AND FLOUR PRODUCTS	293	0	0.00%	275	0	0.00%	-	-	-	10	0	0.00%	-	-	-
6 CONFECTIONERY AND PASTRY PRODUCTS	12,042	49	0.41%	7252	11	0.15%	70	0	0.00%	26	0	0.00%	10	0	0.00%
9 NUTS, INCLUDING PEANUTS	10	0	0.00%	10	0	0.00%	-	-	-	-	-	-	-	-	-
10 VEGETABLES, INCLUDING LEGUMES	2626	1	0.04%	2570	0	0.00%	910	5	0.55%	5	0	0.00%	-	-	-
11 FRUITS	2495	0	0.00%	1275	0	0.00%	537	0	0.00%	-	-	-	-	-	-
16 MINERAL WATERS AND NONALCOHOLIC BEVERAGES	-	-	-	-	-	-	1539	0	0.00%	-	-	-	-	-	-
18 OILSEEDS	5	0	0.00%	5	0	0.00%	-	-	-	-	-	-	-	-	-
19 GROUP FOOD CONCENTRATES	5	0	0.00%	5	0	0.00%	-	-	-	-	-	-	-	-	-
20 MAYONNAISES, MUSTARDS, SAUCES	5	0	0.00%	3	0	0.00%	3	0	0.00%	5	0	0.00%	-	-	-
21 HERBS, SPICES	2225	11	0.49%	2105	0	0.00%	-	-	-	-	-	-	-	-	-
22 COFFEE, TEA, COCOA, FRUIT TEAS AND HERBAL	315	0	0.00%	5	0	0.00%	-	-	-	-	-	-	-	-	-
23 DELICATESSEN AND CULINARY PRODUCTS	2941	13	0.44%	2747	23	0.84%	55	0	0.00%	210	2	0.95%			
24 FOODS FOR SPECIAL NUTRITIONAL USES	3737	0	0.00%	3619	0	0.00%	8	0	0.00%	2	0	0.00%	10	0	0.00%
26 DIETARY SUPPLEMENTS	721	0	0.00%	711	0	0.00%	6	0	0.00%	1	0	0.00%	-	-	-
30 OTHER	482	15	3.11%	85	0	0.00%	90	1	1.11%	270	5	1.85%	-	-	-
TOTAL	43,603	215	0.49%	33,263	39	0.12%	4150	16	0.39%	2229	7	0.31%	214	20	9.35%

**Table 2:** Detailed Results of Monitoring and Official Control Studies Conducted on the Retail Market in Poland in 2017.

	2017														
	*Salmonella* spp.			*L. monocytogenes*			*E. coli*			Coagulase-Positive Staphylococci (Including *S. aureus*)			*Campylobacter* spp.		
PRODUCT GROUP	Tested Samples	Disqualified Samples	Percent of Disqualified Samples	Tested Samples	Disqualified Samples	Percent of Disqualified Samples	Tested Samples	Disqualified Samples	Percent of Disqualified Samples	Tested Samples	Disqualified Samples	Percent of Disqualified Samples	Tested Samples	Disqualified Samples	Percent of Disqualified Samples
1 MEAT, OFFAL AND MEAT PRODUCTS	2866	32	1.12%	1968	0	0.00%	623	1	0.16%	59	0	0.00%	142	11	7.75%
2 POULTRY, OFFAL AND POULTRY PRODUCTS, EGGS AND EGG PRODUCTS	4196	95	2.26%	595	0	0.00%	-	-	-	221	0	0.00%	83	13	15.66%
3 FISH, SEAFOOD AND THEIR PRODUCTS	1559	0	0.00%	1548	5	0.32%	10	0	0.00%	10	0	0.00%	-	-	-
4 MILK AND DAIRY PRODUCTS	8006	0	0.00%	8490	1	0.01%	472	15	3.18%	1455	0	0.00%	-	-	-
5 CEREAL GRAINS AND CEREAL AND FLOUR PRODUCTS	380	0	0.00%	320	0	0.00%	-	-	-	-	-	-	-	-	-
6 CONFECTIONERY AND PASTRY PRODUCTS	12,572	26	0.21%	7264	12	0.17%	18	0	0.00%	11	0	0.00%	-	-	-
10 VEGETABLES, INCLUDING LEGUMES	2367	2	0.08%	2704	0	0.00%	2115	0	0.00%	20	0	0.00%	-	-	-
11 FRUITS	2546	2	0.08%	1370	0	0.00%	1032	0	0.00%	5	0	0.00%	-	-	-
16 MINERAL WATERS AND NONALCOHOLIC BEVERAGES	-	-	-	-	-	-	1570	0	0.00%	-	-	-	-	-	-
18 OILSEEDS	-	-	-	-	-	-	5	0	0.00%	5	0	0.00%	-	-	-
20 MAYONNAISES, MUSTARDS, SAUCES	3	0	0.00%	3	0	0.00%	-	-	-	3	0	0.00%	-	-	-
21 HERBS, SPICES	2231	18	0.81%	2190	0	0.00%	6	0	0.00%	-	-	-	-	-	-
22 COFFEE, TEA, COCOA, FRUIT TEAS AND HERBAL	288	0	0.00%	3	0	0.00%	-	-	-	-	-	-	-	-	-
23 DELICATESSEN AND CULINARY PRODUCTS	2731	0	0.00%	2804	0	0.00%	50	1	2.00%	146	0	0.00%	-	-	-
24 FOODS FOR SPECIAL NUTRITIONAL USES	3541	0	0.00%	3480	0	0.00%	-	-	-	1	0	0.00%	-	-	-
25 NOVEL FOODS	95	0	0.00%	95	0	0.00%	-	-	-	313	0	0.00%	-	-	-
26 DIETARY SUPPLEMENTS	605	0	0.00%	600	0	0.00%	-	-	-	-	-	-	-	-	-
30 OTHER	406	34	8.37%	96	0	0.00%	62	0	0.00%	-	-	-	2	0	0.00%
TOTAL	44,392	209	0.47%	33,530	18	0.05%	5963	17	0.29%	2249	0	0.00%	227	24	10.57%

**Table 3:** Detailed Results of Monitoring and Official Control Studies Conducted on the Retail Market in Poland in 2018.

	2018														
	*Salmonella* spp.			*L. monocytogenes*			*E. coli*			Coagulase-Positive Staphylococci (Including *S. aureus*)			*Campylobacter* spp.		
PRODUCT GROUP	Tested Samples	Disqualified Samples	Percent of Disqualified Samples	Tested Samples	Disqualified Samples	Percent of Disqualified Samples	Tested Samples	Disqualified Samples	Percent of Disqualified Samples	Tested Samples	Disqualified Samples	Percent of Disqualified Samples	Tested Samples	Disqualified Samples	Percent of Disqualified Samples
1 MEAT, OFFAL AND MEAT PRODUCTS	2353	26	1.10%	1590	1	0.06%	465	9	1.94%	15	0	0.00%	65	4	6.15%
2 POULTRY, OFFAL AND POULTRY PRODUCTS, EGGS AND EGG PRODUCTS	3513	158	4.50%	494	0	0.00%	1	0	0.00%	180	0	0.00%	119	28	23.53%
3 FISH, SEAFOOD AND THEIR PRODUCTS	1109	0	0.00%	1122	5	0.45%	1	0	0.00%	2	0	0.00%	-	-	-
4 MILK AND DAIRY PRODUCTS	5729	0	0.00%	6041	0	0.00%	346	0	0.00%	1213	0	0.00%	-	-	-
5 CEREAL GRAINS AND CEREAL AND FLOUR PRODUCTS	525	0	0.00%	240	0	0.00%	-	-	-	-	-	-	-	-	-
6 CONFECTIONERY AND PASTRY PRODUCTS	8556	24	0.28%	5335	2	0.04%	5	0	0.00%	7	0	0.00%			
9 NUTS, INCLUDING PEANUTS	1	0	0.00%	-	-	-	-	-	-	-	-	-	-	-	-
10 VEGETABLES, INCLUDING LEGUMES	1723	10	0.58%	1809	15	0.83%	1635	15	0.92%	9	0	0.00%	-	-	-
11 FRUITS	1692	1	0.06%	865	0	0.00%	505	0	0.00%	6	0	0.00%	-	-	-
16 MINERAL WATERS AND NONALCOHOLIC BEVERAGES	-	-	-	-	-	-	1157	1	0.09%	-	-	-	-	-	-
18 OILSEEDS	199	8	4.02%	-	-	-	-	-	-	-	-	-	-	-	-
19 FOOD CONCENTRATES	10	0	0.00%	-	-	-	-	-	-	-	-	-	-	-	-
21 HERBS, SPICES	1470	6	0.41%	1455	0	0.00%	5	0	0.00%	-	-	-	-	-	-
22 COFFEE, TEA, COCOA, FRUIT TEAS AND HERBAL	240	0	0.00%	-	-	-	-	-	-	-	-	-	-	-	-
23 DELICATESSEN AND CULINARY PRODUCTS	1999	0	0.00%	1887	5	0.26%	16	0	0.00%	106	1	0.94%	-	-	-
24 FOODS FOR SPECIAL NUTRITIONAL USES	2017	0	0.00%	1987	0	0.00%	5	0	0.00%	5	0	0.00%	-	-	-
26 DIETARY SUPPLEMENTS	455	0	0.00%	450	0	0.00%	-	-	-	-	-	-	-	-	-
30 OTHER	269	3	1.12%	69	0	0.00%	97	0	0.00%	100	9	9.00%	1	0	0.00%
TOTAL	31,860	236	0.74%	23,344	28	0.12%	4238	25	0.59%	1643	10	0.61%	185	32	17.30%

**Table 4:** Detailed Results of Monitoring and Official Control Studies Conducted on the Retail Market in Poland in 2019.

	2019														
	*Salmonella* spp.			*L. monocytogenes*			*E. coli*			Coagulase-Positive Staphylococci (Including *S. aureus*)			*Campylobacter* spp.		
PRODUCT GROUP	Tested Samples	Disqualified Samples	Percent of Disqualified Samples	Tested Samples	Disqualified Samples	Percent of Disqualified Samples	Tested Samples	Disqualified Samples	Percent of Disqualified Samples	Tested Samples	Disqualified Samples	Percent of Disqualified Samples	Tested Samples	Disqualified Samples	Percent of Disqualified Samples
1 MEAT, OFFAL AND MEAT PRODUCTS	3683	62	1.68%	2023	0	0.00%	640	18	2.81%	13	0	0.00%	97	1	1.03%
2 POULTRY, OFFAL AND POULTRY PRODUCTS, EGGS AND EGG PRODUCTS	4930	211	4.28%	735	0	0.00%	5	0	0.00%	285	0	0.00%	197	65	32.99%
3 FISH, SEAFOOD AND THEIR PRODUCTS	1861	0	0.00%	1861	1	0.05%	6	0	0.00%	6	0	0.00%	5	0	0.00%
4 MILK AND DAIRY PRODUCTS	8868	5	0.06%	8311	0	0.00%	450	14	3.11%	1780	0	0.00%	-	-	-
5 CEREAL GRAINS AND CEREAL AND FLOUR PRODUCTS	5	0	0.00%	-	-	-	-	-	-	-	-	-	-	-	-
6 CONFECTIONERY AND PASTRY PRODUCTS	11,814	17	0.14%	7797	5	0.06%	29	0	0.00%	40	5	12.50%	-	-	-
10 VEGETABLES, INCLUDING LEGUMES	2554	1	0.04%	3229	14	0.43%	2332	0	0.00%	5	0	0.00%	-	-	-
11 FRUITS	2395	4	0.17%	1756	0	0.00%	776	0	0.00%	1	0	0.00%	-	-	-
16 MINERAL WATERS AND NONALCOHOLIC BEVERAGES	-	-	-	-	-	-	1519	0	0.00%	-	-	-	-	-	-
17 VEGETABLE FATS	10	0	0.00%	5	0	0.00%	-	-	-	-	-	-	-	-	-
18 OILSEEDS	305	5	1.64%	-	-	-	-	-	-	-	-	-	-	-	-
19 FOOD CONCENTRATES	6	0	0.00%	170	0	0.00%	-	-	-	-	-	-	-	-	-
20 MAYONNAISES, MUSTARDS, SAUCES	11	0	0.00%	240	0	0.00%	5	0	0.00%	5	0	0.00%	-	-	-
21 HERBS, SPICES	2302	43	1.87%	2335	0	0.00%	18	0	0.00%	-	-	-	-	-	-
22 COFFEE, TEA, COCOA, FRUIT TEAS AND HERBAL	305	0	0.00%	5	0	0.00%	-	-	-	-	-	-	-	-	-
23 DELICATESSEN AND CULINARY PRODUCTS	3210	1	0.03%	2887	5	0.17%	26	1	3.85%	92	0	0.00%	-	-	-
24 FOODS FOR SPECIAL NUTRITIONAL USES	2877	0	0.00%	2912	0	0.00%	1	0	0.00%	61	0	0.00%	-	-	-
26 DIETARY SUPPLEMENTS	696	0	0.00%	701	0	0.00%	-	-	-	-	-	-	-	-	-
30 OTHER	405	11	2.72%	133	0	0.00%	39	0	0.00%	226	0	0.00%	-	-	-
TOTAL	46,237	360	0.78%	35,100	25	0.07%	5846	33	0.56%	2514	5	0.20%	299	66	22.07%

**Table 5:** Detailed Results of Monitoring and Official Control Studies Conducted on the Retail Market in Poland in 2020.

	2020														
	*Salmonella* spp.			*L. monocytogenes*			*E. coli*			Coagulase-Positive Staphylococci (Including *S. aureus*)			*Campylobacter* spp.		
PRODUCT GROUP	Tested Samples	Disqualified Samples	Percent of Disqualified Samples	Tested Samples	Disqualified Samples	Percent of Disqualified Samples	Tested Samples	Disqualified Samples	Percent of Disqualified Samples	Tested Samples	Disqualified Samples	Percent of Disqualified Samples	Tested Samples	Disqualified Samples	Percent of Disqualified Samples
1 MEAT, OFFAL AND MEAT PRODUCTS	1946	36	1.85%	1067	0	0.00%	505	6	1.19%	-	-	-	-	-	-
2 POULTRY, OFFAL AND POULTRY PRODUCTS, EGGS AND EGG PRODUCTS	2872	124	4.32%	480	2	0.42%	10	6	60.00%	323	0	0.00%	131	29	22.14%
3 FISH, SEAFOOD AND THEIR PRODUCTS	1031	0	0.00%	1026	1	0.10%	1	0	0.00%	115	0	0.00%	-	-	-
4 MILK AND DAIRY PRODUCTS	5659	54	0.95%	5638	59	1.05%	60	5	8.33%	1266	10	0.79%	-	-	-
5 CEREAL GRAINS AND CEREAL AND FLOUR PRODUCTS	1479	0	0.00%	947	0	0.00%	2	0	0.00%	2	0	0.00%	-	-	-
6 CONFECTIONERY AND PASTRY PRODUCTS	5085	9	0.18%	5000	1	0.02%	25	12	48.00%	5	0	0.00%			
10 VEGETABLES, INCLUDING LEGUMES	1741	9	0.52%	2416	4	0.17%	715	0	0.00%	-	-	-	-	-	-
11 FRUITS	1715	5	0.29%	1570	0	0.00%	275	5	1.82%	-	-	-	-	-	-
16 MINERAL WATERS AND NONALCOHOLIC BEVERAGES	-	-	-	-	-	-	873		0.00%	-	-	-	-	-	-
17 VEGETABLE FATS	15	0	0.00%	-	-	-	-	-	-	-	-	-	-	-	-
18 OILSEEDS	245	0	0.00%	-	-	-	-	-	-	-	-	-	-	-	-
19 FOOD CONCENTRATES	35	0	0.00%	-	-	-	-	-	-	-	-	-	-	-	-
21 HERBS, SPICES	1416	25	1.77%	1395	20	1.43%	-	-	-	-	-	-	-	-	-
22 COFFEE, TEA, COCOA, FRUIT TEAS AND HERBAL	120	2	1.67%	-	-	-	-	-	-	-	-	-	-	-	-
23 DELICATESSEN AND CULINARY PRODUCTS	3657	0	0.00%	3630	10	0.28%	20	0	0.00%	11	0	0.00%	-	-	-
24 FOODS FOR SPECIAL NUTRITIONAL USES	2017	0	0.00%	1437	0	0.00%	-	-	-	2	0	0.00%	-	-	-
26 DIETARY SUPPLEMENTS	440	0	0.00%	430	0	0.00%	5	0	0.00%	-	-	-	-	-	-
30 OTHER	242	1	0.41%	96	0	0.00%	7	0	0.00%	28	0	0.00%	-	-	-
TOTAL	29,715	265	0.89%	25,132	97	0.39%	2498	34	1.36%	1752	10	0.57%	131	29	22.14%

## Data Availability

The original contributions presented in this study are included in the article. Further inquiries can be directed to the corresponding author.
